# Identification of a Novel Methylated Gene in Nasopharyngeal Carcinoma: TTC40

**DOI:** 10.1155/2014/691742

**Published:** 2014-06-30

**Authors:** Wajdi Ayadi, Nesrine Allaya, Hanèn Frikha, Emna Trigui, Abdelmajid Khabir, Abdelmonem Ghorbel, Jamel Daoud, Mounir Frikha, Ali Gargouri, Raja Mokdad-Gargouri

**Affiliations:** ^1^Laboratory of Biomass Valorization and Protein Production in Eukaryotes, Center of Biotechnology of Sfax, University of Sfax, 3038 Sfax, Tunisia; ^2^University Hospital Habib Bourguiba of Sfax, 3029 Sfax, Tunisia

## Abstract

To further explore the epigenetic changes in nasopharyngeal carcinoma (NPC), methylation-sensitive arbitrarily primed PCR was performed on NPC biopsies and nontumor nasopharyngeal samples. We have shown mainly two DNA fragments that appeared to be differentially methylated in NPCs versus nontumors. The first, defined as hypermethylated, corresponds to a CpG island at the 5′-end of the tetratricopeptide repeat domain 40 (TTC40) gene, whereas the second, defined as hypo-methylated, is located on repetitive sequences at chromosomes 16p11.1 and 13.1. Thereafter, the epigenetic alteration on the 5′-TTC40 gene was confirmed by methylation-specific PCR, showing a significant aberrant methylation in NPCs, compared to nontumors. In addition, the bisulfite sequencing analysis has shown a very high density of methylated cytosines in C15, C17, and X666 NPC xenografts. To assess whether TTC40 gene is silenced by aberrant methylation, we examined the gene expression by reverse transcription-PCR. Our analysis showed that the mRNA expression was significantly lower in tumors than in nontumors, which is associated with 5′-TTC40 gene hypermethylation. In conclusion, we found that the 5′-TTC40 gene is frequently methylated and is associated with the loss of mRNA expression in NPCs. Hypermethylation of 5′-TTC40 gene might play a role in NPC development; nevertheless, other studies are needed.

## 1. Introduction

Nasopharyngeal carcinoma (NPC) is a malignancy with a remarkable racial and geographic distribution [1, 2]. In Tunisia, it represents the most frequent head and neck cancer, with an annual incidence of about 4 cases per 100,000 persons. There are several clinical and biological characteristics which are specific to North African patients, especially the two peaks of frequency according to age, the first around 50 and the second below 25 (20–25% of cases) [3].

The etiology of NPC is multifactorial and includes genetic susceptibility, exposure to various carcinogens, and Epstein-Barr virus (EBV) latent infection [4, 5]. Epigenetic changes also play a crucial role in nasopharyngeal carcinogenesis. Such epigenetic alterations of malignant tumors, compared with their analogous normal tissues, include both decreases in global DNA methylation (hypomethylation) commonly assessed in DNA repeat regions and concomitant increases (hypermethylation) in specific regions with the ability to affect gene expression [6]. Promoter hypermethylation has been proposed as a common mechanism for the transcriptional inactivation of several tumor suppressor genes (TSGs) in human carcinomas. For NPC, silencing by promoter methylation has been reported for variety of TSGs, including Ras association domain family 1A (RASSF1), retinoic acid receptor *β*2 (RAR *β*2), death-associated protein kinase (DAP-kinase), and deleted in lung and esophageal cancer 1 (DLEC1) [7–9]. It has been demonstrated that the gene alterations are involved in NPC pathogenesis by disrupting the normal regulation of apoptosis and cell cycle control [10–13].

To identify the changes of the CpG methylation profile in cancer development, many methods have been described including those that explore the ability of exclusive digestion of nonmethylated CpG dinucleotides, such as restriction landmark genomic scanning (RLGS) [14], methylated CpG island amplification, followed by representational difference analysis (MCA/RDA) [15], and methylation-sensitive arbitrarily primed PCR (MS/AP-PCR) [16]. In the present study, we used MS/AP-PCR method to explore the CpG methylation profiles in NPC patients and then to identify the DNA fragments that may be differentially methylated in tumors versus nontumor samples. We report, mainly, a frequently methylated CpG island at 10q26.3 locus that is correlated with transcriptional silencing of a previously unknown gene, namely, the tetratricopeptide repeat domain 40 (TTC40).

## 2. Materials and Methods

### 2.1. Patients and Tissue Samples

Primary NPC biopsies were collected with informed consent from 68 patients, prior to any treatment, at Sfax University Hospital, Tunisia. Fifteen cases were of the juvenile form (patients aged less than 30 years). The clinical stage of the disease was determined according to the tumor, node, and metastasis (TNM) classification of the American Joint Committee on Cancer/International Union Against Cancer (AJCC/UICC, 1997) [17, 18]. The histological type of NPC was determined on tissue sections according to the World Health Organization (WHO, 2005) [19]. In addition, twelve biopsies of nontumor nasopharyngeal mucosa were used as controls. All samples were immediately frozen in liquid nitrogen, embedded in optimal cutting temperature (OCT) compound, and subsequently stored at −80°C until use. Hematoxylin and eosin stained sections were examined by pathologists and shown to contain at least 70% of tumor cells.

### 2.2. MS/AP-PCR Conditions

Genomic DNA was isolated using TRIzol reagent (Invitrogen) according to the manufacturer's instructions and conserved at −20°C until use. Methylation-sensitive arbitrarily primed PCR (MS/AP-PCR) was conducted according to Gonzalgo et al. [16], with some modifications. Briefly, 1 *μ*g of DNA in 40 *μ*L was digested with either 10 units of methylation-sensitive restriction enzyme* Hpa*II (Fermentas) or 10 units of methylation-insensitive isoschizomer* Msp*I (Fermentas) at 37°C for 16 h. Aliquot of restriction-digested DNA (4 *μ*L) was amplified in a final volume of 50 *μ*L containing 1x PCR buffer, 50 pmol of a single primer MLG2 (5′-AACCCTCACCCTAACCCCGG-3′), 200 *μ*M of each dNTP, 2 mM of MgCl_2_, and 1.25 units of Taq DNA polymerase (Fermentas). The reactions were carried out in a thermal cycler (Applied Biosystems 2720) for five cycles under low stringency (94°C for 30 s, 40°C for 60 s, and 72°C for 90 s) followed by 30 cycles under high stringency (95°C for 15 s, 55°C for 15 s, and 72°C for 60 s). The PCR products were resolved on 8% polyacrylamide gel and staining was made using silver nitrate, as previously described by Benbouza et al. [20].

### 2.3. Isolation and Sequencing of AP-PCR Fragments

Candidate differentially methylated bands were cut out from dried polyacrylamide gels and incubated in 50 *μ*L of sterile water at 80°C for 10 min. Five microliters of the eluate was reamplified using the same primer MLG2, under the same conditions as described above. The amplified products were resolved on 2% agarose gel stained with ethidium bromide and cloned in a pGEM-T vector(Promega). Individual colonies were subjected to automated sequencing with an ABI 377 DNA autosequencer (Applied Biosystems). Sequence identities were obtained using the BLAST program of the National Cancer Center for Biotechnology Information (http://www.ncbi.nlm.nih.gov/BLAST/).

### 2.4. Bisulfite Sequencing

DNA samples from the three NPC xenografts (C15, C17, and X666) and the MCF-7 breast cancer cell line were subjected to bisulfite sequencing analysis. One *μ*g of genomic DNA was modified by bisulfite treatment for 12 hours using EZ DNA Methylation kit (Zymo Research) according to the manufacturer's instructions. This treatment deaminates unmethylated cytosines into uracil but does not affect 5-methylcytosines. After bisulfite treatment, PCR amplification was performed on 100 ng of modified DNA in a final reaction mixture of 100 *μ*L containing 1x PCR buffer, 1 *μ*M of each primer (Table 1), 200 *μ*M of each dNTP, 2 mM of MgCl_2_, and 2.5 units of Taq DNA polymerase (Fermentas). The amplified fragments were purified using the Wizard SV gel and PCR Clean-Up System (Promega) and cloned into a pGEM-T vector (Promega). Three clones of each sample were subjected to automated sequencing.

### 2.5. MSP Conditions

The methylation status of the 5′-region of TTC-40 gene in the NPC xenografts and biopsies was also examined by two-stage nested methylation-specific PCR (MSP). The primers of the stage-1 PCR recognize the bisulfite-modified template but do not discriminate between methylated and unmethylated sequences. Aliquot of 50 ng of modified DNA was amplified in a final reaction mixture of 25 *μ*L containing 1x PCR buffer, 0.4 *μ*M of each primers, 200 *μ*M of each dNTP, 2 mM of MgCl_2_, and 1.25 units of Taq DNA polymerase (Fermentas). Then, the PCR products were diluted 100-fold and 2 *μ*L was subjected to a stage-2 PCR with two different mix reactions containing primers specific to methylated and unmethylated templates, respectively. Finally, the PCR products were separated on a 2% agarose gel, visualized by ethidium bromide staining. The primer sequences, annealing temperatures, and PCR product sizes are listed in Table 1.

### 2.6. Reverse Transcription- (RT-) PCR

RT-PCR was performed to examine the expression of TTC40 and endogenous control *β*-actin. Total RNA was isolated from frozen tissues using TRIzol [21]. First strand cDNA synthesis was performed on 1 *μ*g of total RNA, previously treated with DNase (Amersham Biosciences), in a final volume of 20 *μ*L containing 1× reaction buffer (Invitrogen), 0.1 pmole of random hexamer primers, 10 nmole of each dNTP, and 100 units of MMLV reverse transcriptase (Invitrogen). The reaction mixture was incubated at 37°C for 1 h, followed by 70°C for 10 min. The cDNA (1 *μ*L) was used as a template in PCR using specific primers for TTC40 (exons 5-6, under Accession number NM_001200049.2) and *β*-actin. The primer sequences, product sizes, and annealing temperatures are shown in Table 1.

### 2.7. Statistical Analyses

Statistical analyses were performed by Fisher's test. A *P* value ≤ 0.05 was considered as statistically significant.

## 3. Results

### 3.1. Differentially Methylated DNA Fragments

**Figure 1:**
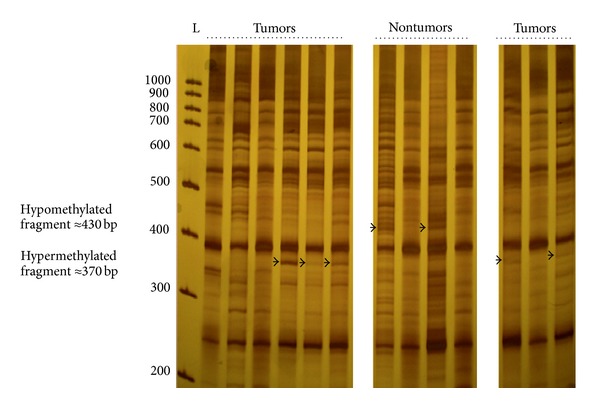
Methylation fingerprints of NPC tumors and nontumor nasopharyngeal tissues using primer MLG2 in the arbitrarily primed-PCR reactions. Bands that appeared to be differentially methylated are indicated by arrows. L: 100 bp DNA ladder.

We used MS/AP-PCR to isolate and identify novel DNA fragments that are aberrantly methylated in NPC tumors (*n* = 18) versus nontumor nasopharyngeal tissues (*n* = 8). Our data showed mainly 2 bands that appeared to be differentially methylated between tumor and nontumor samples. One band of about 370 bp was visualized in 8 out of 18 tumor samples and was considered as a hypermethylated DNA fragment in NPC. The second band of about 430 bp detected in only nontumor samples (*n* = 4) was regarded as hypomethylated DNA fragment (Figure 1).

**Figure 2:**
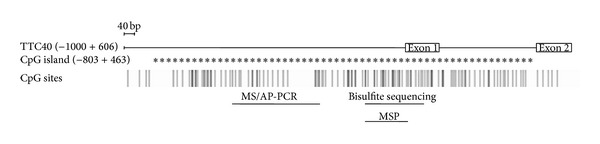
Diagram of CpG island of 5′-TTC40 gene which indicates the position of DNA regions identified by MS/AP-PCR, bisulfite DNA sequencing, and MSP methods. Asterisks (∗) indicate the CpG island of 1267 bp. Vertical line represents a single CpG site. −xxx + xxx relative to the transcription start site.

To identify the differential amplification products, corresponding bands were excised, reamplified, cloned, and subject to DNA sequencing. In fact, our analysis of the hypermethylated fragment revealed two different DNA sequences (Table 2). Among them, we identified a region of 334 bp on chromosome 10q26.3, within a potentially CpG island and exactly at 379 bp upstream, the first exon of TTC40 gene. The CpG island was identified in 5′-flanking of TTC40 gene covering the region −803 to +463, relative to the transcription start site (Figure 2). Regarding the hypomethylated DNA fragment, we found similar DNA sequences of 400 or 406 bp in size. They are located on repetitive genomic region at chromosomes 16p11.1 and 13.1, respectively (Table 2). For the remainder of this study, we focused on the TTC40 gene analysis: status of methylation and expression in NPC samples.

### 3.2. TTC40 Methylation Status

**Figure 3:**
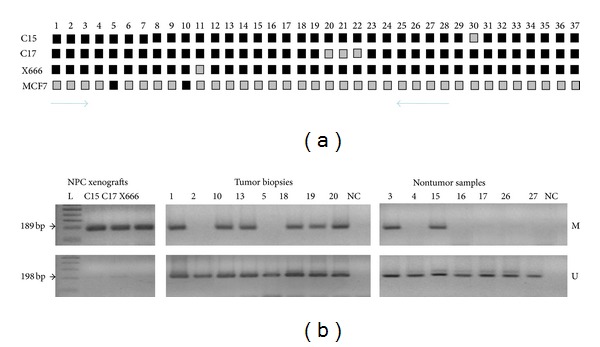
(a) Sequencing results of 5-TTC40 gene from three NPC xenografts (C15, C17, and X666) and the MCF-7 breast cancer cell line. The methylation status of CpG is indicated by shading: black (methylated) and grey (partially methylated). The arrows indicate the position of primers used by MSP. (b) MSP results of 5′-TTC40 gene from the three NPC xenografts and the representative cases of tumor biopsies and nontumors samples. NC: negative control (sterile distilled water). M and U represent the amplification of methylated and unmethylated sequences, respectively. L: 100 bp DNA ladder.

To verify the MS/AP-PCR results, we performed the bisulfite genomic sequencing and MSP on NPC samples. The schematic position of the analyzed DNA sequences on the 5′-CpG island of TTC-40 gene is shown in Figure 2. Bisulfite DNA sequencing was conducted to determine the methylation pattern of a 294 bp region containing 37 CpG sites in three NPC xenografts (C15, C17, and X666). This region has no significant similarity with mouse genome by blast program and therefore the expected results should reflect the methylation status in only human origin cells. In fact, our analysis was focused on three clones and based on these data, each CpG site was classified as methylated, partially methylated, or unmethylated. As shown in Figure 3(a), the 5′CpG island of TTC40 gene had a very high density of methylated cytosines in all NPC xenografts. In contrast, most of these CpG sites were partially methylated in the MCF-7 breast cancer cell line. Next, we applied a MSP method to assess the frequency of the aberrant methylation of the 5′-CpG island of TTC40 gene in 45 primary NPC biopsies and 11 nontumor nasopharyngeal tissues. Among the NPC biopsies, none was fully methylated, 32 were partially methylated (71.12%), and 13 were unmethylated. For the nontumor tissues, only 2 were partially methylated (18.19%) and the remaining were unmethylated (Figure 3(b)). The high frequency of TTC40 gene methylation in NPC cases compared to nontumor cases was statistically significant (*P* = 0.002). We note that the fully methylated form was shown in only NPC xenografts, which is in agreement with bisulfite genomic sequencing data.

To determine whether the aberrant methylation of TTC40 gene is correlated with clinicopathological parameters of patients with NPC, the frequency rates of the methylated status was calculated according to the bimodal-age distribution, histology type, tumor size, lymph node involvement, and distant metastasis. The statistical results have not shown any significant association (data not shown).

### 3.3. TTC40 Aberrant Methylation Was Correlated with Transcriptional Silencing

**Figure 4:**
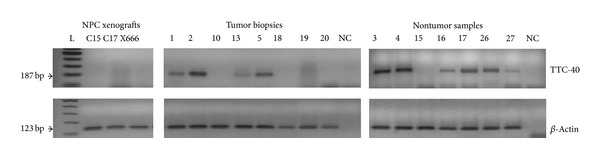
RT-PCR results of TTC40 gene from the three NPC xenografts and the representative cases of tumor biopsies and nontumors samples. NC: negative control (sterile distilled water). *β*-Actin was used as an endogenous control. L: 100 bp DNA ladder.

To assess whether the transcriptional repression of TTC40 gene is associated with the aberrant CpG methylation, we examined its corresponding mRNA expression in tumors and nontumor tissues. Our RT-PCR results showed that the TTC40 gene was silenced in all three NPC xenografts and in more than fifty percent of primary NPC cases (31/55; 56.37%). However, the TTC40 expression was commonly detected in nontumor nasopharyngeal tissues (6 out of 7 cases; 85.72%) (Figure 4). The low frequency of TTC40 expression in NPC cases compared to nontumor cases was statistically significant (*P* = 0.042). Among all the specimens assessed by RT-PCR for TTC-40 expression, only 41 available cases were tested for methylation status: three NPC xenografts, 32 primary tumors, and 6 nontumors (Table 3). In these cases, a significant correlation was found between aberrant CpG methylation and transcriptional silencing of TTC-40 gene (*P* = 0.004) (Table 4).

## 4. Discussion

Genome-wide analysis for identification of hypermethylated regions in several tumor tissues has been reported and it leads to the isolation of novel or known cancer-related genes. Here, we identified 2 novel DNA fragments differentially methylated in NPC tumors compared to normal nasopharyngeal tissues. To our knowledge, this is the first study that focuses on MS/AP-PCR analysis to explore the CpG methylation profiles in NPC patients. Nevertheless, MS/AP-PCR method has previously been used for the identification of differentially methylated DNA in a variety of cancer such as colon, breast, prostate, and esophagus [16, 22–25]. In fact, novel cancer-related genes that are epigenetically silenced have been described and, thereafter, defined as important molecular actors in the development of malignant diseases, especially the T-box transcription factor 5 (TBX5) gene in colon cancer [25] and the pituitary tumor apoptosis gene (PTAG) in pituitary adenomas [26].

Our finding shows that the isolated hypermethylated fragment by MS/AP-PCR corresponds to a CpG island located at the 5′-end of a previously unknown gene, TTC40. The recommended name, TTC40, has been attributed recently to the detection of its full transcript of 8293 nucleotides, under Accession number NM_001200049. The corresponding protein was not experimentally provided and it remains as hypothetical product. It was predicted to be 2715 aa showing 13 tetratricopeptide repeat (TPR) patterns (Link Q8IYW2, UniProtKB/Swiss-Prot). The TPR pattern was originally identified in yeast as a module of protein-protein interaction involved in the regulation of the cell cycle [27]. Here, our result on TTC40 gene would mention a possible relationship of its aberrant methylation resulting in its transcriptional silencing in NPC tumors. The methylation status of the 5′-TTC40 gene was also conducted by the bisulfite sequencing of 37 CpG sites, showing a very high density of methylated cytosines in three NPC xenografts C15, C17, and X666. In addition, the MSP result shows a high frequency of aberrant methylation in 32 out of 45 primary NPC tumors (71.12%). It was significantly more common compared to normal nasopharyngeal tissues (*P* = 0.002) which is in line with the data on MS/AP-PCR. Overall, our data could suggest the methylation of 5′-TTC40 gene as a putative NPC biomarker since this epigenetic change is significantly more frequent in tumor than in normal nasopharyngeal mucosa. On the other hand, we consider that the TTC40 methylation frequency was similar to those previously found for the most defined genes as epigenetically silenced in NPC patients such as RASSF1A, RAR *β*2, DAP-kinase, and DLEC1 [7–10]. Consequently, we should support the inclusion of this epigenetic alteration in the panel of molecular biomarkers for primary diagnosis and monitoring of patients with NPC. Based on the general assumption about the impact of CpG island methylation on gene silencing [6, 28], we expected that this epigenetic alteration might play important roles in transcriptional repression of TTC40 in NPC tumors. In this regard, we found that the aberrant methylation was strongly correlated with the loss of TTC40 expression in forty-one available cases that were tested by both MSP and RT-PCR (*P* = 0.004). The current data may suggest that this epigenetic event is responsible, at least in part, for the silencing of TTC40 in NPC malignancy. However, further studies are warranted on the transcriptional regulation of TTC40 gene in NPC by analyzing, for example, the effect of DNA-demethylating agent on the TTC40 gene expression.

The second identified fragment by MS/AP-PCR corresponds to hypomethylated DNA sequence in NPC tumors. It does not meet the CpG islands criteria, likely reflecting the general DNA hypomethylation that occurs at non-CpG-rich regions in tumor cells [29]. In addition, the DNA sequence is located in repetitive genomic regions at the short arm of chromosome 16 that is prone to a number of recurrent rearrangements due to the presence of flanking segmental duplications. Recently, it has been shown that the recurring imbalances are associated with abnormal phenotypes including autism, intellectual disabilities, behavioural disorders, congenital anomalies, and obesity [30, 31]. However, these genetic alterations are not previously reported in malignant diseases. Therefore, the possible effect of the epigenetic change of chromosomes 16p11.1 and 13.1 on cancer development should be verified in further researches.

## 5. Conclusion 

In summary, our results support the search of novel DNA fragments exhibiting aberrant methylation in NPC. Indeed, we mainly identified the 5′-CpG island of TTC40 gene as a new frequent cancer-associated hypermethylation in patients with NPC. This epigenetic event is suggested as a molecular factor involved in the loss of TTC40 expression. These findings promote the starting of further molecular investigations to examine the potential role of the epigenetic inactivation of TTC40 gene in the tumorigenesis of NPC and other cancers.

## Figures and Tables

**Table 1:** Summary of primers used for MSP, bisulfite sequencing, and RT-PCR.

Primer sequences (5′-3′)	Annealing temperatures (°C)	Product sizes (bp)	PCR cycles
**MSP** “TTC40”			
*Stage-1 PCR *			
F-GAAGGTGGAGGGTGAGTT	52	439	25
R-AACCTAAAATCACCTTACTAACTCT			
*Stage-2 PCR *			
**M**F-ATTTTCGCGGAGTTGGCG	61	189	35
**M**R-CTCCGAAATCCGCGATACG			
**U**F-GTTTTATTTTTGTGGAGTTGGTG	59	198	35
**U**R-CTCTCTCCAAAATCCACAATACA			

**Bisulfite sequencing** “TTC40”			
F-GTTGTTTTGGTGTTTTATTTT	50	294	35
R-AACCTAAAATCACCTTACTAACTCT			

**RT-PCR**			
“TTC40”			
F-CCAGCCTTTCCCAAATCATA	61	187	40
R-ATCTGCCGGTATTTCTGTGG			
“*β*-Actin”			
F-GTCTCCCAAGTCCACACA	57	125	30
R-GCACGAAGGCTCATCATT			

**Table 2:** Summary of the two major differentially methylated DNA fragments identified by MS/AP-PCR.

Fragment	Size (bp)	CpG dinucleotide	CpG island^a^	Unique/repetitive	Chromosome map
**Hypermethylated**					
Sequence-1	334	25	Yes	Unique	10q26.3
Sequence-2	336	3	No	Unique	1p13.3

**Hypomethylated**					
Sequence-1	400	7	No	Repetitive (6x)	16p11.1
Sequence-2^b^	406	8	No	Repetitive (6x)	16p13.1

^a^The presence of CpG island was determined based on the following criteria: minimum length, 200 bp; CG content, >50%; Obs/Exp, >0.6.

^b^That is different from sequence-1 by the insertion of 6 bp.

**Table 3:** Series of clinical specimens analyzed by MSP and/or RT-PCR.

	MSP only	MSP and RT-PCR	RT-PCR only	Total
Primary NPC biopsies	13	32	23	68

Nontumor samples	5	6	1	12

Total	18	38	24	

**Table 4:** Relationship between TTC40 methylation status and its expression.

		Methylation status			*P* value
		M (*n* = 3^a^)	M/U (*n* = 24^b^)	U (*n* = 14^c^)	
mRNA expression	+ (*n* = 23^d^)	0	11	12	0.004
	− (*n* = 18^e^)	3	13	2	

M: full methylation, M/U: partial methylation, and U: unmethylation.

a: 3 NPC xenografts, b: 23 tumor biopsies + 1 nontumor, c: 9 tumor biopsies + 5 nontumors; d: 18 tumor biopsies + 5 nontumors; e: 3 NPC xenografts + 14 tumor biopsies + 1 nontumor. *P* value based on Fisher's test.
